# *Chromobacterium violaceum* Periareolar Infection, First Non-Lethal Case in Colombia: Case Report and Literature Review

**DOI:** 10.3390/idr13020053

**Published:** 2021-06-21

**Authors:** Diego Alejandro Cubides Diaz, Daniel Arsanios Martin, Nicolas Bernal Ortiz, Ana Lucia Ovalle Monroy, Valentina Hernandez Angarita, Yesid Fabian Mantilla Florez

**Affiliations:** 1 Department of Internal Medicine, Universidad de La Sabana, Chía 140013, Colombia; danielmaar@unisabana.edu.co (D.A.M.); yesidmanfl@unisabana.edu.co (Y.F.M.F.); 2 Faculty of Medicine, Universidad de La Sabana, Chía 140013, Colombia; nicolasbeor@unisabana.edu.co (N.B.O.); anaovmo@unisabana.edu.co (A.L.O.M.); valentinahean@unisabana.edu.co (V.H.A.)

**Keywords:** *Chromobacterium violaceum*, periareolar infection, soft tissue infection

## Abstract

*Chromobacterium violaceum* is a facultative anaerobic, Gram-negative rod found in different ecosystems, especially tropical and subtropical areas. Human infections are rare, and just a few cases have been reported in literature. In this paper, we present the first non-lethal infection due to *Chromobacterium violaceum,* in an adult male with polycystic kidney disease in Colombia. Periareolar soft tissue infection was documented with isolation of *Chromobacterium violaceum*. Clinical manifestations, treatment, and outcome are shown.

## 1. Introduction

*Chromobacterium violaceum* is a facultative anaerobic, Gram-negative rod of the *Neisseriaceae* family that is considered an opportunistic pathogen. It is mainly found in water and soil; transmission is by contact with organic material and (to a lesser extent) by contaminated water ingestion [1,2]. Mortality rates range between 65 and 80% [2]. Until 2021, 200 cases had been reported worldwide, with two in Colombia—one of them in a child [3] and the latter in an adult [4], both with fatal outcomes. In this paper, we present the first non-fatal soft tissue infection due to *Chromobacterium violaceum* in an adult with polycystic kidney disease in Colombia, with a favorable outcome.

## 2. Case Report

A 47-year-old man (an employee of the hotel sector from Bogotá) with a past medical history of polycystic kidney disease presented to the emergency room with complaints of one week of erythema and purulent drainage of the anterior chest wall associated with a fever of up to 38.5 °C. He received cephalexin during a three-day course without any improvement.

**Figure 1:**
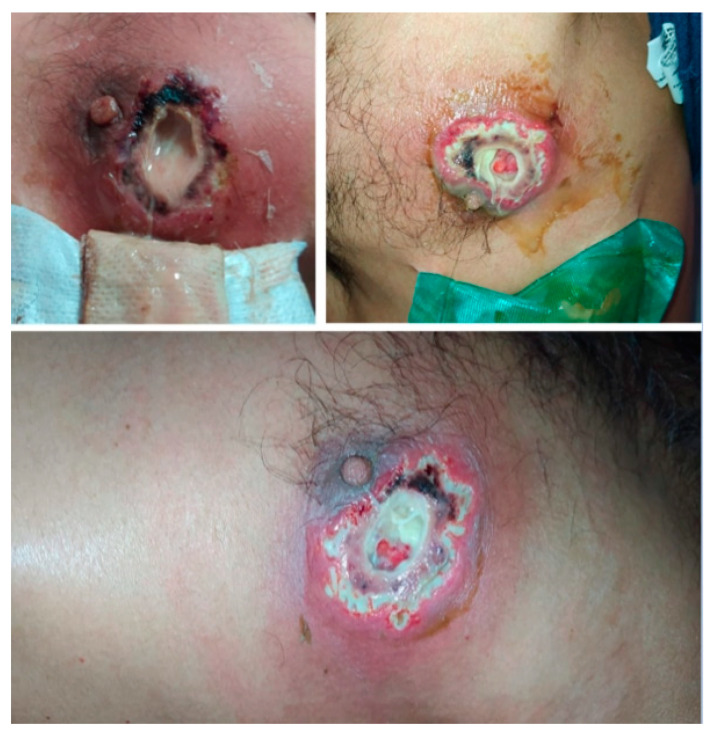
Periareolar lesion with erythematous borders and purulent discharge.

Upon physical examination, he was in no acute distress, and his skin exam was remarkable for an erythematous left periareolar nodule with swelling, warm, and purulent discharge (Figure 1), along with signs of systemic inflammatory response without clinical findings of sepsis organic dysfunction (Table 1). The patient started on therapy with intravenous clindamycin. However, he deteriorated due to the worsening of chronic kidney disease with the further requirement of renal replacement therapy.

**Figure 2:**
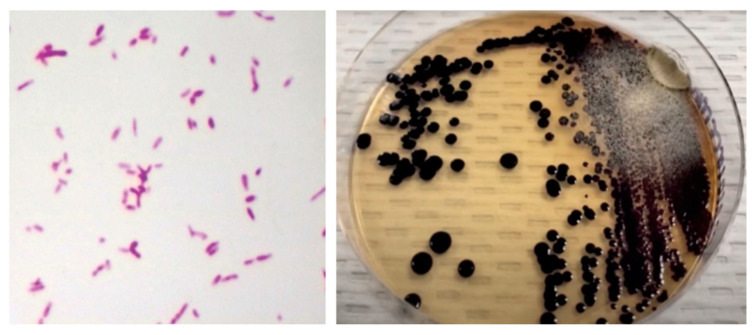
(**Left**) Gram stain of skin abscess showing Gram-negative coccobacillary forms. (**Right**) nutritive agar with black-violet mucoid colonies consistent with *Chromobacterium violaceum*.

The patient was taken to surgery, where a 15-cm abscess with skin necrosis over the area of renitence was found. The cytochemical results of the abscess showed abundant leukocytes and the isolation of *Chromobacterium violaceum* (Figure 2). Antibiograms were not made, and blood cultures were negative. The patient continued intravenous clindamycin with no improvement at the third day of admission, so doxycycline was started at a 100 mg BID dosage with partial response. At day five of admission, he was taken to another surgical procedure, and negative pressure therapy was placed over his wound. This therapy allows one to draw fluid from an infected site using a vacuum system, which is especially useful for deep, complicated, and nonhealing wounds. The antibiotic was again changed to tigecycline at a 50 mg BID dosage for ten days.

**Figure 3:**
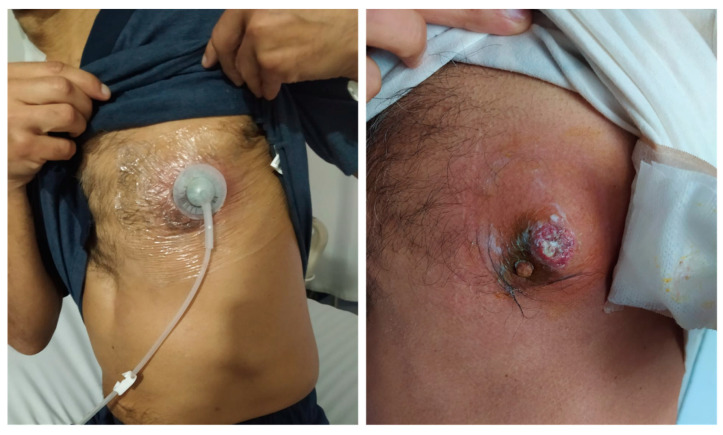
Lesion evolution at discharge, after antibiotic and negative pressure therapy.

After five days of tigecycline, he underwent surgery again, negative pressure therapy was withdrawn, and the wound was covered with a fasciocutaneous flap. He was discharged after completing 10 days of antibiotic treatment, with no signs of systemic inflammatory response and with adequate closure of the skin wound (Figure 3).

## 3. Discussion

*Chromobacterium violaceum* is a facultative anaerobic, carbohydrate fermenter, catalase-positive, and Gram-negative bacillus. It grows on blood, chocolate, and MacConkey agar within 18–24 h and can produce pigmented colonies due to the production of violacein, a metabolite with apoptotic activity on some cells [5]. Nonetheless, it can also grow as unpigmented colonies.

This microorganism adheres through the CopE membrane protein, which induces rearrangement in the actin cytoskeleton, thus leading to damage in epithelial cells. Furthermore, metalloproteinase (MMP-2) with elastase activity facilitates the production of abscesses, and two type III secretion systems, which are involved in the signal transduction cascade that favor cytotoxicity by allowing for pore formation in host cell membranes, have been described [5].

Skin and soft tissue infections due to this Gram-negative bacteria mainly occur in patients with risk factors—either environment-related, such as exposure to organic or contaminated surfaces (especially if there is a loss of continuity of the skin) or host-related, such as chronic immunosuppression conditions like diabetes mellitus, chronic granulomatous disease (CGD), and glucose-6-phosphate dehydrogenase (G6PD) deficiency [6].

There are two ways to explain the susceptibility to *Chromobacterium violaceum* infections in patients with these last two conditions: The first is related to the dysfunction of the phagocytic cell to eliminate the bacteria, since the production of peroxide is altered due to the deficiency of NADPH and NADPH oxidase. The hydrogen peroxide necessary for phagocytic activity comes from two routes: first after the action of lysosomal enzymes and acid hydrolases, and second through the action of the enzyme NADPH oxidase. Catalase-positive microorganisms such as *Chromobacterium violaceum* disable the production of lysosomal hydrogen peroxide, leaving only the NADPH route in charge of its production for phagocytic action. Thus, in conditions like CGD and G6PD deficiency where NADPH production is compromised, there is a major susceptibility to infections due to these microorganisms [7]. The second mechanism refers to the inadequate formation of the inflammasome; it has been reported that NADPH pathway is directly involved with the regulation of the latter, so any alteration in the NADPH production (as in CGD and G6PDH) would directly affect the destruction of inflammasome-dependent microorganisms, such as *Burkholderia cepacia* and *Chromobacterium violaceum* [8,9,10]. 

The association of *Chromobacterium violaceum* infection with CGD has been previously described, mainly in children since the hereditary nature of the disease and almost none in adults with skin and soft tissue infections [11,12,13,14,15,16,17,18,19,20,21,22,23,24,25,26,27]. Nonetheless, this association does not seem to be directly related to an increase in mortality [28]. Fewer cases of infections in patients with G6PDH deficiency have been reported, perhaps because of the rarity of the disease, so it is not possible to set a mortality trend in this condition [29,30]. In our patient, the only triggering factor corresponded to an insect bite, which had been previously reported in two cases [12,22]. 

To date, 27 cases of skin and soft tissue infection by *Chromobacterium violaceum* have been reported in adults (Table 2), mainly described in males of ages between 20 and 50 years old, with an average of 46 years. Most of them have presented with an acute onset of symptoms, frequently requiring hospitalization in the first week [16,19,20,22,25,27]: 44% of infections have presented with bacteremia and 51% have led to sepsis, with mortality as high as 22%. Bacterial growth in culture has ranged from 15 to 72 h, except for a single reported case of 10 days. In most cases, the definitive treatment was found to be a beta-lactam and fluoroquinolone combination, consistent with most reported susceptibility profiles where resistance to penicillins, aminopenicillins, and cephalosporins, as well as sensitivity to carbapenems and fluoroquinolones, is frequent (Table 2).

The patient in this case presented a severe clinical course, with organ dysfunction and with requirement of multiple surgical procedures to control the infectious source. The patient had an inappropriate response to initial antimicrobial therapy, which is consistent with previous reported cases where broad spectrum antibiotic combinations were required for control of the infection. However, in comparison with the two other reported cases in Colombia, both of them with fatal outcomes, he did not present bacteremia during his clinical course. In fact, bacteremia and sepsis were present in all of the reviewed cases of skin and soft tissue infections with fatal outcomes, suggesting that the dissemination of the infection into the bloodstream is a strong predictor of adverse outcomes and mortality.

In Colombia, this is the first case of non-lethal infection by *Chromobacterium violaceum*, and it was the first one of skin and soft tissue infection (Table 3). In Latin America, it corresponds to the first case of non-lethal skin and soft tissue infection [18]

## 4. Conclusions

*Chromobacterium violaceum* has been reported since 1927 as the cause of severe infections, with a trend towards immunocompromised populations and patients of reproductive age with skin trauma. Thus far, the inflammasome and NADPH pathway dysfunction are the most studied predisposing factors for infection, while the presence of bacteremia is a strong predictor of adverse outcomes and mortality. The mechanisms of antibiotic resistance are not yet clear, but carbapenems and fluoroquinolones are the most widely used antibiotics in hospitalized patients. 

Many factors contributed to clinical cure in the presented case, including an adequate control of infectious source and tissue debridement, the absence of bloodstream infection, and a strong antibiotic therapy. More studies are required to understand the dynamics of this microorganism and its clinical implications. 

## Figures and Tables

**Table 1:** Laboratory findings.

Laboratory	Admission	Discharge
White blood cell count	22.03 × 10^3^/µL	11.92 × 10^3^/µL
Neutrophils	18.9 × 10^3^/µL (85.9%)	6.6 × 10^3^/µL (55.2%)
Lymphocytes	1.33 × 10^3^/µL (6%)	3.56 × 10^3^/µL (29.9%)
Monocytes	1.61 × 10^3^/µL (7.3%)	1.18 × 10^3^/µL (9.90%)
Red blood cells	3.89 × 10^6^/µL	3.35 × 10^6^/µL
Hematocrit	33.3%	29%
Hemoglobin	11.2 g/dL	9.70 g/dL
MCV	85.6 fl	86.6 fl
MCH	28.8 pg	29 pg
MCHC	33.6 g/dL	33.4 g/dL
RDW	12.7%	12.5%
Platelets	264 × 10^3^/µL	407 × 10^3^/µL
C-reactive protein	176.4 mg/L	12.29 mg/L
Blood urea nitrogen	89.7 mg/dL	74.3 mg/dL
Creatinine	9.14 mg/dL	5.16 mg/dL

**Table 2:** *Chromobacterium violaceum* skin and soft tissue infections reported worldwide.

Author/Year	Age and Gender	Clinical Manifestations	Related Condition	Time to Positive Culture	Bacteremia	Sepsis	Resistance Profile	Antibiotic Treatment	Outcome
Sneath et al. [11] (1953)	Not reported (M)	Left thigh ulcer with inguinal lymphadenopathy fever and hepatomegaly.	Not reported.	24 h	Yes	Yes	Not reported	TCY	Septic shock and multi-organ dysfunction. Death 26 days after admission.
Kaufman et al. [12] (1986)	44 yo (F)	Ulcerated nodules and purpura in the abdomen, limbs, and back. Fever, abdominal pain, hepatomegaly, and jaundice.	Exposure to contaminated water. Wasp sting.	24 h	Yes	Yes	Sensitive: MZL, GEN, CLO, TCY, CMX CBN, NMC, and NTM Resistant: PEN, AMP, AMK, TOB, PMX B, and CFS	MEZ and GEN	Septic shock. Death at 6 weeks after admission.
Georghiou et al. [13] (1989)	35 yo (M)	Posterior neck wound. Twelve days later: extension to the right shoulder with abscess, abdominal pain, fever, and diarrhoea.	Abrasion while carrying old damp floor-covering on that shoulder	24 h	Yes	Yes	Sensitive: GEN, CIP, and CLO, IPM, TOB, and AMC Resistant: ATM, AMX, SXT, and CEP	GEN, IPM, and CIP	Alive, fully recovered.
Huffman et al. [14] (1998)	46 yo (M)	Three weeks of malaise, hyporexia, nausea, abdominal pain, vomiting, 7 kg loss, left thigh wound, and liver abscess.	Heavy smoking.	24 h	Yes	Yes	Sensitive: GEN, CIP, CLO, TCY, IPM, and SXT Resistant: CRO, AMC, and CAZ	CLO, GEN, and TCY	Resolved. Neuropathy as a sequel of the disease.
Huffman et al. [14] (1998)	53 yo (M)	7 mm wound on the sole of the left foot; six days later: fever, pain, and purulent discharge.	Diabetes mellitus. Wound with rusty metal.	Not reported	No	No	Sensitive: GEN, CIP, IPM, PIP, TOB, and AMC Resistant: CAZ	AMC, GEN, DOX, and CIP	Alive, fully recovered.
Dan et al. [15] (2001)	24 yo (M)	Pimple in right cheek with purulent discharge for three weeks. Fever, headache, and dizziness for 2 weeks. Brain abscess was reported.	Farm laboring.	24 h	No	No	Sensitive: IPM and CIP Resistant: CTX and CRO	CIP	Alive, fully recovered.
Hee kim et al. [1] (2005)	38 yo (M)	Polytrauma with multiple wounds, rib fractures, empyema, hemothorax, tibial fracture, kidney, and liver hematoma.	Hit by a car while fishing. Contaminated water exposure.	Not reported	No	No	Sensitive: CIP, AMK, GEN, TZP, LVX, and SXT, Resistant: CRO, AMP, TOB, SAM, and FEP	CFA and AZM	Alive, fully recovered.
Teoh et al. [16] (2006)	40 yo (M)	1 cm forearm abscess. One day later: purulent discharge epigastric pain and fever. Peritonitis was reported.	Forearm wound during camping near a lagoon.	Not reported	Yes	Yes	Not reported	CIP, MDZ, and CTX	Septic shock. Death at 20 h after admission.
Lim et al. [17] (2009)	40 yo (F)	Anterior chest wall wound. Malaise, fever, chills, lumbar pain, and headache. Apical pansystolic murmur, liver abscess, and vegetations on the coronary cusps.	Wound with a tree branch during lake swimming.	28–39 h	Yes	Yes	Sensitive: CIP, TZP, and MEM, IPM, SXT, and CFX Resistant: AMP, CXM, and CAZ	MEM and CIP	Liver abscess and infective endocarditis resolved after 6–11 weeks, respectively. Alive.
Yang et al. [18] (2011)	64 yo (M)	Right foot erythematous lesion with pain and edema. Headache and fever.	Diabetes mellitus and hepatitis B infection. Fish bite in a river.	10 days	Yes	Yes	Sensitive: CIP, TZP, and MEM, LVX, ATM, CAS, and FEP Intermediate: GEN and AMK Resistant: SAM	CRO, CIP, and DOX	Alive, fully recovered.
Kumar et al. [19] (2012)	42 yo (M)	Head and leg lesions. Seven days later: pain, swelling, and purulent discharge.	Exposure to contaminated water.	24 h	No	No	Sensitive: GEN, CIP, and AMK, CLO, TCY, CAZ, and IPM Intermediate: CTX Resistant: PEN and CFX	GEN	Alive, fully recovered.
Ansari et al. [20] (2015)	45 yo (M)	Wound in the middle finger of the left hand. Seven days later: fever, epigastric pain, swelling, and purulent discharge.	Stinging wound with unknown object.	12 h	Yes	Yes	Sensitive: AMK, GEN, CIP, TZP, MEM, CRO, SXT, AMC, MDZ, and FLX.	TZP, FLX, and MDZ.	Septic shock and death 21 h after admission.
Madi et al. [21] (2015)	53 yo (F)	Left leg lesion. Two weeks later: abdominal pain and vomiting; the lesion then ulcerated. Hepatomegaly and jaundice.	Skin trauma during farm laboring.	Not reported	Yes	Yes	Sensitive: CIP, TZP, MEM IPM, SXT, and CFX Resistant: AMP, CXM, and CAZ	IPM, CIP, and TZP	Alive, fully recovered.
Lin et al. [22] (2016)	23 yo (F)	Limb injury. Eight months later: granulation tissue in the wound.	Wound contamination in muddy field.	Not reported	No	No	All isolates were sensitive to CIP, MEM FEP, and GEN	TZP	Alive, fully recovered.
Lin et al. [22] (2016)	42 yo (F)	Chest wall wound.	Not reported	Not reported	Yes	Yes		TZP, MEM, and GEN	Sepsis, mesenteric ischemia, abdominal abscess and fat necrosis. Deceased.
Lin et al. [22] (2016)	53 yo (F)	Limb wound, diabetic foot with infection, and abscess.	Diabetes mellitus	Not reported	No	No		GEN and SXT	Alive, fully recovered.
Lin et al. [22] (2016)	52 yo (M)	Post-surgical infected sternotomy wound.	Myocardial revascularization	Not reported	Not reported	Yes		TZP	Alive, fully recovered.
Lin et al. [22] (2016)	32 yo (M)	Lacerating injury on the toes with purulent discharge.	Wound during swamp hike.	Not reported	No	No		SXT	Alive, fully recovered.
Lin et al. [22] (2016)	57 yo (M)	Anterior chest wall wound.	Found unconscious in a swamp.	Not reported	Not reported	Not reported		MEM	Alive, fully recovered.
Lin et al. [22] (2016)	45 yo (M)	Wound on the palm of the hand.	Stab wound. Possible contaminated metal.	Not reported	No	No		DOX	Alive, fully recovered.
Lin et al. [22] (2016)	50 yo (M)	Wound on toe	Not reported	Not reported	No	No		CZO and DOX	Alive, fully recovered.
Lin et al. [22] (2016)	21 yo (M)	Stinging wound with abscess in upper limb.	Spider bite.	Not reported	No	No		FLX and AMC	Alive, fully recovered.
Matsuura et al. [23] (2017)	69 yo (F)	Internal malleolus wound in right leg, fever, and liver abscess.	Rice cultivation and contaminated water.	72 h	Yes	Yes	Sensitive: LVX, GEN, TCY, and SXT Resistant: All betalactams.	CIP	Alive, fully recovered.
Sachu et al. [24] (2020)	76 yo (F)	Several pustulous skin lesions predominantly in the right forearm; intermittent fever.	Myelodysplastic syndrome, autoimmune hemolytic anemia, diabetes mellitus type 2, typhoid fever, and recurrent urinary tract infection. Exposure to contaminated water.	15 h	Yes	Yes	Sensitive: AMK, GEN, CIP, MEM, and LVX Resistant: AMP, AMC, and CFS	MEM	Septic shock, acute kidney failure and death 72 h later.
Khadanga et al. [25] (2020)	62 yo (M)	Left foot ulcer with necrosis associated with five days of abdominal pain and hepatomegaly.	Diabetes mellitus/	8 h	Yes	Yes	Sensitive: CIP, GEN, AMK, TZP, TCY, CAZ, FEP, IPM, NTM, and GFX. Resistant: AMP, AMC, and CTX	CRO, VAN, and CIP	Alive, fully recovered.
Mazumder et al. [26] (2020)	40 yo (M)	Left ankle contusion. Fifteen days later: fever, chills, abdominal pain, sweating, and purulent discharge from the wound.	Contusion in rice crop fields and contaminated water exposure.	12 h	No	No	Sensitive: AMK, AZM, CIP, NIT, TZP, MEM, IPM, LVX, ETP, SXT, GEN, TOB, and NTM Resistant: CRO, AMP, CAZ, CTX, AMC, COL, PMX B, and CFM	MEM and CIP	Alive, fully recovered.
Zhang et al. [27] (2021)	50 yo (M)	Left leg wound. Seven days later: ulceration, bleeding, purulent discharge, and necrosis.	Wound while laboring in the field.	24 h	No	No	Sensitive: TZP, CIP, AMK, MEM, LVX, IPM, TGC, and CFP Resistant: AMP, PEN, and CFS	TZP and LVX	Alive, fully recovered.

AMK: amikacin; AZM: azithromycin; CIP: ciprofloxacin; NIT: nitrofurantoin; TZP: piperacillin/tazobactam; CLI: clindamycin; GEN: gentamicin; MEM: meropenem; CRO: ceftriaxone; TCY: tetracycline; CAZ: ceftazidime; FEP: cefepime; IPM: imipenem; AMP: ampicillin; AMC: amoxicillin/clavulanate; CTX: cefotaxime; PIP: piperacillin; AMX: amoxicillin; CEP: cephalothin; NTM: netilmicin; CFM: cefixime; CFX: cefoperazone/sulbactam; MZL: mezlocillin; PEN: penicillin; CFS: cephalosporins; CMX: cotrimoxazole; CBN: carbenicillin; GFX: gatifloxacin; CZO: cefazolin; DOX: doxycycline; MEZ: mezlocillin; CFA: cefpodoxime acid; CTN: cefoxitin; OXA: oxacillin; LVX: levofloxacin; ATM: aztreonam; SAM: ampicillin/sulbactam; SXT: trimethoprim/sulfamethoxazole; VAN: vancomycin; CXM: cefuroxime; ETP: ertapenem; MDZ: metronidazole; CLO: chloramphenicol; COL: colistin; TOB: tobramycin; PMX B: polymyxin B; TGC: tigecycline; FLX: flucloxacillin.

**Table 3:** *Chromobacterium violaceum* infections reported in Colombia.

Author	Year	Age	Gender	Bacteremia	Resistance Profile	Antibiotic Treatment	Outcome
Díaz J, et al. [22]	2007	38	M	Yes	Sensitive: IPM, MEM, and TZP Resistant: GEN, AZM, AMK, CRO, FEP, CTX, CAZ, CIP, and PIP	CIP, CRO, and OXA	Death
Mattar S, et al. [23]	2007	4	M	Yes	Sensitive: CLO, TCC, SXT, and CIP Resistant: AMP, CFS, CTN, CRO, CTX, CAZ, ATM, IPM, AMK, GEN, and TOB	CRO, AMK, and CIP	Death

AMK: amikacin; AZM: azithromycin; CIP: ciprofloxacin: TZP: piperacillin/tazobactam; GEN: gentamicin; MEM: meropenem; CRO: ceftriaxone; TCY: tetracycline; CAZ: ceftazidime; FEP: cefepime; IPM: imipenem; AMP: ampicillin; AMC: amoxicillin/clavulanate; CTX: cefotaxime; PIP: piperacillin; CFS: cephalosporins; CTN: cefoxitin; OXA: oxacillin; ATM: aztreonam; SXT: trimethoprim/sulfamethoxazole; TOB: tobramycin; PMX B: polymyxin B; TGC: tigecycline; FLX: flucloxacillin.

## Data Availability

All relevant information has been presented in the case report. Any additional data may be made available on reasonable request from the corresponding author.
